# An updated view of the pathogenesis of steroid-sensitive nephrotic syndrome

**DOI:** 10.1007/s00467-021-05401-4

**Published:** 2022-01-10

**Authors:** Tomoko Horinouchi, Kandai Nozu, Kazumoto Iijima

**Affiliations:** 1 Department of Pediatrics, Kobe University Graduate School of Medicine, Kobe, Japan; 2 Hyogo Prefectural Kobe Children’s Hospital, Kobe, Japan; 3 Department of Advanced Pediatric Medicine, Kobe University Graduate School of Medicine, Minatojimaminami-machi 1-6-7, Chuo-ku, Kobe, 650-0047 Japan

**Keywords:** Steroid-sensitive nephrotic syndrome, Immune system, HLA class II, Podocyte disorders, *NPHS1*, Autoantibody

## Abstract

Idiopathic nephrotic syndrome is the most common childhood glomerular disease. Most forms of this syndrome respond to corticosteroids at standard doses and are, therefore, defined as steroid-sensitive nephrotic syndrome (SSNS). Immunological mechanisms and subsequent podocyte disorders play a pivotal role in SSNS and have been studied for years; however, the precise pathogenesis remains unclear. With recent advances in genetic techniques, an exhaustive hypothesis-free approach called a genome-wide association study (GWAS) has been conducted in various populations. GWASs in pediatric SSNS peaked in the human leukocyte antigen class II region in various populations. Additionally, an association of immune-related *CALHM6/FAM26F*, *PARM1*, *BTNL2*, and *TNFSF15* genes, as well as *NPHS1*, which encodes nephrin expressed in podocytes, has been identified as a locus that achieves genome-wide significance in pediatric SSNS. However, the specific mechanism of SSNS development requires elucidation. This review describes an updated view of SSNS pathogenesis from immunological and genetic aspects, including interactions with infections or allergies, production of circulating factors, and an autoantibody hypothesis.

## Introduction

Idiopathic nephrotic syndrome (NS) is the most common childhood glomerular disease. The incidence of this syndrome has been reported to be 1.15–16.9/100,000 children, and it is highest in non-Western countries [1]. Most of these patients are initially treated with corticosteroids and fall into one of the following two broad categories: steroid-sensitive nephrotic syndrome (SSNS) and steroid-resistant nephrotic syndrome (SRNS), in which corticosteroids induce and do not induce remission, respectively [2]. In SRNS, abnormalities in podocyte-associated genes have been identified in approximately 30% of patients [3–5], and the mechanism of pathogenesis associated with structural abnormalities appears to be the most relevant. Conversely, immunological mechanisms and subsequent podocyte disorders have been considered [1]. Although genetic research, including genome-wide association study (GWAS), has improved the understanding of these mechanisms, the precise pathophysiology of SSNS remains elusive. In this review, we describe an updated view of SSNS pathogenesis from immunological and genetic aspects, including interactions with infections or allergies, production of circulating factors, and an autoantibody hypothesis.

## Immunological aspects of SSNS

### T cell theory

The pivotal role of prednisolone and the efficacy of immunosuppressive agents in SSNS treatment strongly implicate the immune system in the pathogenesis of the disease. The involvement of T cells in nephrotic syndrome (NS) was reported in the 1970s [6]. The main basis of this T cell theory is as follows: (1) there is an absence of routine deposition of immunoglobulins or complement in the glomeruli, suggesting the involvement of humoral factors; (2) immunosuppressants that suppress T cell function (corticosteroids, ciclosporin, and cyclophosphamide) are effective; and (3) some cases achieve remission following measles infection, which impairs T-cell function [7]. After the 1970s, the following relationships between NS and T cells were reported: upregulation of CD8 + cytotoxic T cells with downregulation of CD4 + T helper (Th) cells, an imbalance between Th2 and Th1 cells resulting in Th2 upregulation, and an imbalance between regulatory T cells and Th17 cells resulting in the prevalence of Th17 cells (reviewed in [8]). However, because of the heterogeneity of NS, these results were not replicable and did not account for all of its pathologies.

### B cell theory

Based on the highly effective therapeutic effects of B cell-depleting monoclonal antibody rituximab (RTX) [9, 10], the hypothesis that B cells are associated with disease was proposed in the 2010s. RTX is a chimeric anti-CD20 monoclonal antibody initially developed to treat B-cell non-Hodgkin lymphoma [11]. CD20 is expressed on the surface of all B cells from the pro-B phase until they eventually differentiate into plasma cells. The parallel occurrence of B cell depletion and a decrease in disease activity in NS strongly suggests a direct involvement of B cell pathology [12, 13]. Moreover, the lymphocyte subset most associated with relapse after RTX administration are switched memory B cells [14]. Additionally, mycophenolate mofetil, useful in preventing the recurrence of SSNS, suppresses switched memory B cells dominantly, suggesting that B cells are also involved in its pathogenesis [15]. Another possible mechanism for the efficacy of RTX in controlling NS is its direct action on the glomeruli or T cells. Various immunosuppressive agents act not only on immune cells but also directly on podocytes [16]. RTX also binds to acid sphingomyelinase-like phosphodiesterase 3b, which is expressed in glomerular epithelial cells [17]. However, ofatumumab, a humanized anti-CD20 monoclonal antibody with different corresponding epitopes, is also effective in patients with SSNS [18]. Therefore, the direct effect of RTX is thought to be exerted via B cells. B cells also interact with T cells in general antigen presentation; accordingly, the immune system is extremely intertwined so that both cell types cannot be separated.

### CD80 and human leukocyte antigen class II

**Fig. 1:**
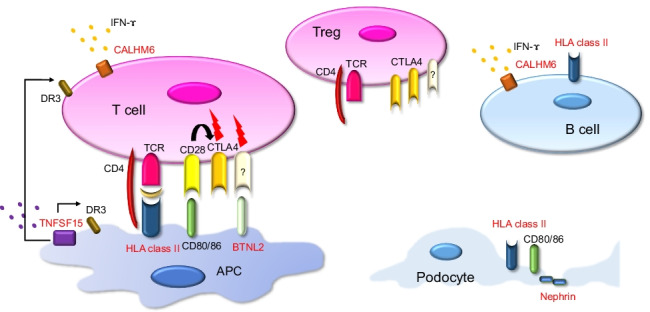
Molecules possibly involved in the pathogenesis of SSNS. The molecules identified in the genome-wide association studies are highlighted in red. HLA class II molecules present antigens mainly in APCs and B cells, but they are also expressed in podocytes. *BTNL2* shares a common structure with CD 80/86 and may be involved in T cell regulation. *CALHM6* is expressed in various lymphocytes and releases cytokines such as IFN-γ. *TNFSF15* interacts with death receptor 3 and activates immune cells. Nephrin is a key component of the slit diaphragm in podocytes. APC, antigen-presenting cell; TCR, T cell receptor; Treg, regulatory T cell; *BTNL2*, butyrophilin-like 2; CTLA4, cytotoxic T-lymphocyte-associated-4; IFN-γ, interferon-gamma; *TNFSF15*, tumor necrosis factor superfamily member 15; DR3, death receptor 3; Flash, some signals related to T cell differentiation

CD80 (B7-1), important for B-cell and T-cell interaction, has attracted considerable attention because of its association with SSNS. CD80 is a transmembrane protein expressed in activated B cells and antigen-presenting cells. During antigen presentation, CD80 binds to CD28 on Th cells or cytotoxic T-lymphocyte-associated-4 on Treg cells and controls the activation or inactivation of T cells [19] (Fig. 1). Although podocytes are highly differentiated glomerular-specific cells, they express CD80 and possess immunogenic aspects [20–24] (Fig. 1). However, it is unclear whether urinary CD80 is useful in differentiating minimal change disease and focal segmental glomerular sclerosis or cytotoxic T-lymphocyte-associated-Ig is effective in refractory NS [25–27]. Podocytes express human leukocyte antigen (HLA) class II and function as immune cells [21]. HLA is a cell-surface molecule that presents specific antigen peptides to the host immune system, such as T cells [28]. Aberrant expression of HLA class II causes autoimmune diseases in antigen-presenting cells and various organs [29]. These facts suggest that podocytes can act as immune cells in the pathogenesis of NS.

### Circulating factors

The pathophysiological role of circulating factors in NS has been suggested, especially in patients with focal segmental glomerulosclerosis (reviewed in [30, 31]). The involvement of circulating factors has been demonstrated by trying to identify a substance from the serum of patients, proving that it induces urinary protein in animal models, and examining the effect on podocytes or endothelial cells, among others. The recurrence of NS in patients with focal segmental glomerulosclerosis after kidney transplantation from unaffected donors strongly suggests the existence of circulating factors [32]. In patients with minimal change disease, interaction with vascular permeability factor derived from T cells or hemopexin has been indicated [31]. Activated hemopexin has been identified in patients with recurrent MCD, and it has also been reported that hemopexin induces transient urinary protein in rats [33, 34]. A certain number of patients are thought to have NS caused by circulating factors [35], but there is no conclusion on how many whose SSNS is attributed to circulating factors or what the specific circulating factor is.

### Interaction with infection

The role of infection as a trigger for the onset or relapse of SSNS has been suggested [36, 37]. Dossier et al. investigated the prevalence of herpes viruses at the onset of childhood NS [38]. They measured the amount of Epstein–Barr virus (EBV), cytomegalovirus (CMV), human herpesvirus 6, and human herpesvirus 7 in peripheral blood and specific antibodies against EBV and CMV. They found that the prevalence of EBV DNA was higher in patients at the onset of NS than in controls.

EBV persists in circulating memory B cells without virion production by establishing latency [39]. How EBV latency affects B cells remains elusive, but it is associated with the risk of developing various autoimmune diseases [40]. EBV downregulates CD74, which may be a way of avoiding HLA class II antigen presentation and consequent CD4 + Th cell recognition while latent in B cells [41]. EBV survival strategies can alter B cells, and their effects can determine SSNS disease susceptibility.

### Interaction with allergy

Many studies have investigated the association between SSNS and allergic diseases such as asthma, atopic dermatitis, or hay fever (reviewed in [42]). Wei et al. reported that patients in the cohort with atopic dermatitis had twice the incidence of NS [43]. Elevated serum IgE levels are known to be a trigger in allergic diseases, and a previous study showed that some patients with NS also have elevated IgE levels [44]. At present, the prevailing view is that common pathogenesis exists between allergic disease and SSNS rather than NS caused solely by allergic diseases [42]. Allergy-causing IgE is produced by B cells, and it is known that allergy is caused by Th2 activation due to Th2/Th1 imbalance; this may coexist with the T cell and B cell theories described above. However, no clear, direct pathology has been established.

## Genetic aspects of SSNS

### Variants in a single causative gene

Advances in genetics have played a major role in the pathogenesis of NS. Approximately 30% of patients with SRNS have a single causative gene associated with podocytes [3–5]. SSNS and SRNS appear to be on the same spectrum and overlap because patients with SSNS may develop steroid resistance during the disease and may partially respond to immunosuppressant therapy, even if genetic abnormalities are identified [45, 46]. A combination of linkage analysis and whole-exome sequencing identified *EMP2* mutations in SSNS [47]. Recently, six genes associated with Rho-like small guanosine triphosphate–binding enzyme activity (*MAGI2*, *TNS2*, *DLC1*, *CDK20*, *ITSN1*, and *ITSN2*) were identified as causes of NS that can partially respond to steroids [48]. However, such variants in a single causative gene account for only a few families in SSNS. Most SSNS cases are considered to be multifactorial, and several reports have clarified susceptibility genes in SSNS [49–53] (reviewed in [54]).

### GWAS for clarifying susceptibility genes

GWAS is a research method for clarifying single-nucleotide polymorphisms (SNPs) related to disease susceptibility genes by comprehensively examining and comparing SNPs as polymorphic markers between case and control groups [55]. Chromosomal recombination is repeated throughout a generation, resulting in genotypes similar to those of adjacent SNPs. Consequently, the genotypes of SNPs nearby tend to have a non-independent distribution in the population, which is called linkage disequilibrium [56]. In GWASs, the use of this property to impute tens of millions of SNPs across the whole genome from hundreds of thousands of SNPs genotyped by microarrays is common [57].

### HLA class II region

Gbadegesin et al., using hypothesis-free exome-wide study methods, first showed that the *HLA-DQ* region was significantly associated with SSNS in children in a South Asian population (Table 1) [49]. The *HLA-DR/DQ* gene encodes an HLA class II molecule required for antigen presentation by antigen-presenting cells or B cells (Fig. 1). In 2018, Jia et al. showed that the most significant association in the *HLA-DR/DQ* region was observed in Japanese childhood SSNS using GWAS methods (Table 1) [52]. In 2018, Debiec et al. also found SSNS-associated SNPs in the *HLA-DR/DQ* region due to a trans-ethnic GWAS from European cohorts (Table 1) [50]. HLA class II regions are highly polymorphic because of their natural selection against various pathogens [58]. Additionally, adaptation to infection has also been reported to contribute to the development of autoimmune diseases including multiple sclerosis and systemic lupus erythematosus [59]. A high polymorphism in the HLA class II region causes difficulty in imputation. Moreover, because *HLA-DQ* and *HLA-DR* regions have a strong linkage disequilibrium relationship, assessing which *HLA-DQ* and *HLA-DR* regions define true disease susceptibility is difficult. Using new HLA imputation methods [60] and genotyping, Jia et al. showed that HLA-DRB1*08:02-DQB1*03:02 was the most significant genetic susceptibility factor [52]. In addition to identifying the relevant SNPs, identifying the actual disease susceptibility alleles may be essential for clarifying the subsequent pathophysiology.

**Table 1:** Genetic aspects of SSNS

Discovery study (case)	Replication study	Trans-ethnic meta-analysis	HLA region	HLA type*	Out of HLA region	Functional analysis	Reference
South Asian (*n* = 214)	N/A	N/A	*HLA-DQ*	N/A	N/A	N/A	Gbadegesin et al.^$^ 2015
Japanese (*n* = 224)	Japanese	N/A	*HLA-DR/DQ*	HLA-DRB1*08:02-DQB1*03:02	N/A	N/A	Jia et al. 2018
European (*n* = 132), African (*n* = 56), Maghrebian (*n* = 85)	European	European, African, Maghrebian	*HLA-DR/DQ*	HLA-DRB1*07:01-DQA1*02:01-DQB1*02:02	*BTNL2*	eQTLs (*HLA*)	Debiec et al. 2018
European (*n* = 422)	N/A	N/A	*HLA-DR/DQ*	HLA-DQA1*02:01	*CALHM6/FAM26F*	eQTLs (*CALHM6*)	Dufek et al. 2019
					*PARM1*		
Japanese (*n* = 987)	Korean, South Asian, African	Japanese, Korean, South Asian, African, European, Hispanic, Maghrebian	*HLA-DR/DQ*	HLA-DRB1*08:02-DQB1*03:02	*NPHS1-KIRREL2*	Allele-specific expression (*NPHS1*)	Jia et al. 2020
					*TNFSF15*	mRNA expression (*TNFSF15*)	

^*^Allele or haplotype with strongest association

^$^Exome-wide association study

Furthermore, expression quantitative trait loci (eQTL) analysis is a powerful tool to investigate the relationship between protein or mRNA expression levels and SNPs. For eQTL analysis, databases such as the international GTEx project and NephQTL specializing in kidney tissue (glomerulus and tubulointerstitium) can be used [61, 62]. Debiec et al. investigated the glomerular eQTL of identified SNPs and found that SSNS-associated SNPs (rs1063348) decreased the expression of *HLA-DRB1*, *HLA-DRB5*, and *HLA-DQB1* [50].

### Outside of the HLA class II region (immune-related)

Dufek et al. reported that, in addition to the strongest association in the *HLA-DR/DQ* region, *CALHM6/FAM26F* and *PARM1* were loci that achieved genome-wide significance (Table 1) [51]. In addition, *BTNL2* and *TNFSF15* were identified by Debiec et al. and Jia et al., respectively, as susceptibility genes for SSNS [50, 53]. *CALHM6/FAM26F* and *PARM1* are related to immunity, even though they are outside the HLA class II region. *BTNL2* encodes the HLA class II-associated transmembrane protein, butyrophilin-like 2 (BTNL2), which is a member of the immunoglobulin superfamily and is implicated as a costimulatory molecule involved in T cell modulation, based on its homology with B7-1 (CD80) (Fig. 1) [63]. Signals mediated by BTNL2 induce FoxP3 and expedite differentiation of naïve T cells into regulatory T cells [64]. BTNL2 is expressed at the highest levels in the intestine and is involved in intestinal immunity [65]. Moreover, *BTNL2* is a disease susceptibility gene for many autoimmune diseases, such as sarcoidosis, ulcerative colitis, systemic lupus erythematosus, and rheumatoid arthritis [66] (reviewed in [63]).

*CALHM6/FAM26F* encodes calcium homeostasis modulator family member 6 (CALHM6), previously called a family with sequence similarity 26, member F (FAM26F) [67]. CALHM6 is a transmembrane protein expressed in various immune cells and plays an important role in diverse immune responses (Fig. 1) [68]. Specific cell–cell interactions and their roles remain unclear but likely to contribute to interferon-γ secretion [69]. The lead SNP (rs2637678) identified by Dufek et al. showed a strong eQTL for *CALHM6*, and the risk allele decreased the expression of *CALHM6* [51]. Although they did not conduct replication analysis, they identified the same SNP (rs2858829) as that identified by Debiec et al. as a marginal genome-wide significant SNP in a region near *CALHM6* [50]. Therefore, the involvement of this region has been demonstrated in multiple datasets.

*TNFSF15* encodes tumor necrosis factor superfamily member 15 (TNFSF15), which interacts with death receptor 3, promotes inflammatory responses in human macrophages, and is associated with apoptosis, cell proliferation, and polarization to Th1 and Th17 cells [70]. Serum TNFSF15 levels are significantly increased in inflammatory bowel disease and primary biliary cirrhosis [71, 72]. Jia et al. discovered a genome-wide significant association in the *TNFSF15* region. One of its replicated SNPs (rs4979462) was also known to be associated with susceptibility to primary biliary cirrhosis, which affected mRNA expression [73]. The mechanism by which these alterations cause NS is unclear, but the immune balance may be altered.

### Outside of the HLA class II region (podocyte-related)

In addition to the *HLA-DR/DQ* region, Jia et al. identified genome-wide significant variants in the *NPHS1-KIRREL2* region (rs56117924) in an extended GWAS in Japanese childhood SSNS [53]. Jia et al. also conducted a replication study, and significant associations were replicated in Korean, South Asian, and African populations [53]. Additionally, a trans-ethnic meta-analysis of Japanese, Korean, South Asian, African, Hispanic, European, and Maghrebian populations showed genome-wide significant associations of variants in the *NPHS1-KIRREL2* region (rs2285450 and rs2073901) [53]. The SNPs rs2285450 (*NPHS1* NM_004646.4: c.294C > T) and rs2073901 (*NPHS1* NM_004646.4: c.2223C > T) are synonymous variants of the *NPHS1* gene in exons 3 and 17, respectively. *NPHS1* encodes nephrin, a molecule located in the slit diaphragm between the foot processes of podocytes (Fig. 1) [74], and is the causative gene of congenital NS Finnish type and SRNS [3–5]. Although there is no evidence that these synonymous variants act as eQTLs, RNA sequencing data allowed the observation of significant allele-specific expression, resulting in lower *NPHS1* expression in haplotypes with risk alleles [53]. Future studies must examine why such allele-specific expression occurs and how it causes SSNS. Findings reported by Jia et al. showed that the gene responsible for a monogenic rare disease (congenital NS Finnish type, SRNS) could be a susceptibility gene for a relatively common multifactorial disease (SSNS). Recently, such a wide range of mutations in a single gene has received much attention [75, 76].

### Functional study

The next challenge is to clarify the significance of disease susceptibility variants detected by GWAS, that is, the direct mechanism causing the disease. For the development of disease-specific therapies, it is highly desirable to elucidate the mechanism. As mentioned above, eQTL analysis about candidate SNPs has become widely used, but an analysis of the relationship between the eQTL and the pathogenesis is needed. Potential methods for further functional analysis include in vitro analysis or analysis using organoids constructed from iPS cells derived from patients (reviewed in [77]). In addition to 3D organoid construction, 2D organoid construction (glomerulus on a chip) is also in progress and could be applied to various related research [77]. In particular, it is often difficult to obtain fresh samples (e.g., kidney biopsy samples) for RNA analysis, which can become an obstacle in clarifying pathogenesis. Therefore, combining such organoid construction technology and RNA-seq analysis may help elucidate SSNS pathogenesis.

## Perspectives

Although the pathogenesis of SSNS has not been clarified, the autoantibody hypothesis has recently been a focus. Many diseases in which HLA class II is identified as a disease susceptibility gene are autoimmune diseases associated with autoantibody [78–80]. In membranous nephropathy, associated with massive proteinuria and which is common in adults, *PLA2R* and *HLA-DQA1* regions are disease susceptibility genes [81]. An autoantibody reactive with the M-type phospholipase A2 receptor encoded by the *PLA2R* gene is associated with membranous nephropathy [82]. As mentioned above, *NPHS1* encoding nephrin is a susceptibility gene; therefore, anti-nephrin antibodies may be involved in some patients with SSNS. In animal models, anti-nephrin antibodies cause NS [83]. Recently, Watts et al. reported that circulating autoantibodies against nephrin were detected in approximately 30% of patients with minimal change disease. The anti-nephrin antibody titer was correlated with disease activity [84]. Moreover, autoantibodies against molecules related to podocytes other than nephrin have been identified in some patients with NS [85, 86]. Conclusively, future research to clarify the pathogenesis of SSNS is expected based on the knowledge derived from hypothesis-free genetic techniques.

## Data Availability

Not applicable.
